# Efficient Bayesian Species Tree Inference under the Multispecies Coalescent

**DOI:** 10.1093/sysbio/syw119

**Published:** 2017-03-06

**Authors:** Bruce Rannala, Ziheng Yang

**Affiliations:** 1 *Department of Evolution and Ecology, University of California, Davis, CA 95616, USA*; 2 *Department of Genetics, Evolution and Environment, University College London, London WC1E 6BT, UK*

## Abstract

We develop a Bayesian method for inferring the species phylogeny under the multispecies coalescent (MSC) model. To improve the mixing properties of the Markov chain Monte Carlo (MCMC) algorithm that traverses the space of species trees, we implement two efficient MCMC proposals: the first is based on the Subtree Pruning and Regrafting (SPR) algorithm and the second is based on a node-slider algorithm. Like the Nearest-Neighbor Interchange (NNI) algorithm we implemented previously, both new algorithms propose changes to the species tree, while simultaneously altering the gene trees at multiple genetic loci to automatically avoid conflicts with the newly proposed species tree. The method integrates over gene trees, naturally taking account of the uncertainty of gene tree topology and branch lengths given the sequence data. A simulation study was performed to examine the statistical properties of the new method. The method was found to show excellent statistical performance, inferring the correct species tree with near certainty when 10 loci were included in the dataset. The prior on species trees has some impact, particularly for small numbers of loci. We analyzed several previously published datasets (both real and simulated) for rattlesnakes and Philippine shrews, in comparison with alternative methods. The results suggest that the Bayesian coalescent-based method is statistically more efficient than heuristic methods based on summary statistics, and that our implementation is computationally more efficient than alternative full-likelihood methods under the MSC. Parameter estimates for the rattlesnake data suggest drastically different evolutionary dynamics between the nuclear and mitochondrial loci, even though they support largely consistent species trees. We discuss the different challenges facing the marginal likelihood calculation and transmodel MCMC as alternative strategies for estimating posterior probabilities for species trees. [Bayes factor; Bayesian inference; MCMC; multispecies coalescent; nodeslider; species tree; SPR.]

Multilocus genetic sequence data have gained importance in inferring species trees in recent years and several inference methods have been proposed for this purpose (Edwards et al. 2016; Xu and Yang 2016, for recent reviews). As noted by Maddison (1997) several processes can cause the species tree to differ from gene trees underlying particular loci. Some of these processes, such as introgression between species and horizontal gene transfer, involve reticulations in the species tree, whereas others, such as incomplete lineage sorting and gene duplications, occur within the context of a nonreticulate (and typically binary) species tree. An important potential source of gene-tree versus species-tree conflicts among genetically isolated species is incomplete lineage sorting, which is typically modeled using a coalescence process.

A simple widely used method for multilocus species tree inference concatenates sequences from different loci, assuming that a single tree (treated as the species tree) underlies all the loci (reviewed in Rannala and Yang 2008; Edwards 2009). This approach can lead to strongly supported incorrect phylogenetic trees when incomplete lineage sorting occurs (see e.g., Leaché and Rannala 2011), and has been shown to be inconsistent (Kubatko and Degnan 2007). Another heuristic approach is to infer separate gene trees and then attempt to reconcile the differences among gene trees to obtain an estimate of the species tree (Page and Charleston 1997). The majority-vote method, which uses the most frequent gene tree among loci as the estimate of the species tree, can be inconsistent when the species tree and parameters are in the so-called “anomaly zone” (Degnan and Salter 2005; Degnan and Rosenberg 2006).

Maddison (1997) and Maddison and Knowles (2006) proposed a parsimony-inspired method for inferring the species tree, called minimizing deep coalescence (MDC) events for gene trees. Other examples include species tree estimation by minimizing coalescence times across genes (the Global LAteSt Split, GLASS; Mossel and Roch 2010), by using the average ranks of coalescences (STAR, Liu et al. 2009) or average gene-tree internode distances (NJst, Liu and Yu 2011), by using average coalescence times (STEAC, Liu et al. 2009), by using maximum likelihood for gene trees under coalescence (STEM, Kubatko et al. 2009), and by maximum pseudo-likelihood (MP-EST, Liu et al. 2010). Similarly ASTRAL (Mirarab and Warnow 2015) finds the species tree that agrees with the largest number of quartet trees induced by the collection of unrooted gene trees. All those methods treat the estimated gene trees (including either the gene tree topology alone or both the gene tree topology and branch lengths) as data, ignoring phylogenetic uncertainties. Such approximations can lead to systematic biases as well as underestimation of the uncertainty of inferred species trees (Leaché and Rannala 2011). The heuristic methods are computationally efficient and can be applied to genome-scale datasets, but they are not statistically efficient (Leaché and Rannala 2011; Liu et al. 2015; Ogilvie et al. 2016).

A parametric statistical method for inferring the species tree using multilocus sequence data should integrate over the unobserved gene trees (both the tree topology and branch lengths). For the case of three species, with one sequence from each species at each locus, a maximum likelihood method used numerical integration to integrate out the two coalescent times in each gene tree (Yang 2002; Dalquen et al. 2016). For larger problems with more species or more sequences, maximum likelihood is not computationally feasible. Instead the Bayesian method is used, with Markov chain Monte Carlo (MCMC) used for the computation. A few MCMC implementations now exist to estimate species trees under the MSC, including BEST (Edwards et al. 2007; Ronquist et al. 2012), *BEAST (Heled and Drummond 2010), BPP (Yang and Rannala 2014), and revBayes (Hohna et al. 2016), although they are limited to a small number of species and loci, and suffer from mixing problems when there are $\gt 100)$ loci, say, in the dataset.

Under the MSC, the gene trees and the species tree impose constraints on each other, which become a serious challenge for designing efficient MCMC algorithms under the model. The divergence time ($t_{AB})$) between two sequences from species $A)$ and $B)$ at any locus must be greater than the divergence time ($\tau_{AB})$) between species $A)$ and $B)$, with $t_{AB} \gt \tau_{AB})$: in other words, *sequences split before species* (see Fig. 1). Such constraints can cause serious difficulties in analysis of large datasets, leading to poor MCMC mixing, when one attempts to change the species tree topology or species divergence times if the gene trees at the mutliple loci are fixed. Two solutions are possible to this difficulty: (i) integrating out the gene trees analytically without the need for MCMC and (ii) developing efficient MCMC proposals to modify the species tree and the gene trees jointly, maintaining the constraint. Recent methods for inferring species trees from single nucleotide polymorphism (SNP) data follow the first strategy (Bryant et al. 2012). The simplicity of these data allow the gene trees to be integrated out of the model analytically. However, a drawback of such methods is that SNPs provide little information about branch lengths in the gene trees and the power may be reduced in comparison with sequence-based methods. The SVDquartets method recently developed by Chifman and Kubatko (2014) takes a similar approach, assuming independence among all sites given the species tree, and calculates the site-pattern probabilities for quartets by integrating out the gene tree topologies and coalescent times analytically.

**Figure 1.**
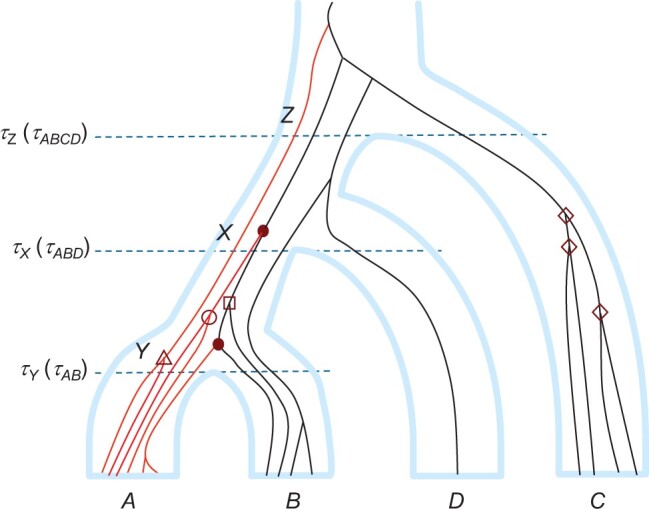
The SPR move makes coordinated changes to the species tree and the gene trees to avoid conflicts between the proposed species tree and the gene trees. The species tree is represented by the light blue boundary pipes while the gene tree is represented by lines running inside the species-tree branches. The SPR move prunes off branch $Y)$-$A)$ on the species tree (including clade $A)$) and reattaches it to a randomly-chosen target branch $C)$, while changing the gene trees through similar SPR moves to avoid conflict. Moved nodes on the gene tree reside in species $AB)$ ($Y)$) or in a species on the path from $Y)$ to $Z)$ (the common ancestor of $A)$ and $C)$), and have exactly one daughter node with descendents in $A)$ only. They are marked by $\bullet)$, and are pruned off and regrafted to a randomly chosen branch on the gene tree that resides in a species on the path from $C)$ to $Z)$. Other affected nodes, marked by $\bigcirc)$, $\triangle)$ or $\Box)$, have their population IDs changed by the move.

Here, we follow the second approach and develop a Bayesian inference procedure for the analysis of multilocus sequence data that jointly infers the species tree and gene trees as well as other relevant parameters such as species divergence times and ancestral population sizes ($\tau)$s and $\theta )$s). We extend our program BPP (for Bayesian Phylogenetics and Phylogeography) (Yang and Rannala 2010; Rannala and Yang 2013; Yang and Rannala 2014) to allow this joint inference. We develop two novel MCMC proposals that change the species tree, at the same time modifying the gene trees to avoid conflicts between the gene trees and newly proposed species tree. The first move is based on the Subtree Pruning and Regrafting (SPR) algorithm for rooted trees. This changes the species tree topology whereas preserving the node ages in the species tree as well as in the gene trees. The second move is based on a node-slider algorithm, which changes the topology as well as the node ages in the species tree and gene trees. Note that the NNI, SPR, and nodeslider moves considered here make coordinated changes to the species tree and to the gene trees at multiple loci. They are far more complex than similar MCMC moves in standard Bayesian phylogenetics programs such as MrBayes or BEAST (Lakner et al. 2008; Hohna et al. 2008; Yang 2014). The two new proposal algorithms lead to considerably improved mixing behavior of the MCMC in comparison with the simple NNI algorithm implemented in our previous work (Yang and Rannala 2014). We also explore the calculation of the marginal likelihood for a given species tree as an approach to comparing alternative species trees under the MSC. We apply our newly developed method to two sets of empirical data, for rattlesnakes and Philippine shrews, respectively.

## Theory

Here we review the formulation of the species tree inference problem in a Bayesian framework and then describe our new MCMC algorithms. Let $X_i)$ be the sequence alignment for locus $i)$. The number of sequences per species may vary for each locus and some species may not be sampled for a particular locus. Our requirement is that every locus should have at least two sequences. Let there be $L)$ loci and define $X = \{ X_i \})$ to be the full dataset. Let $G_i)$ be the gene tree for the sequences sampled at locus $i)$ (including both the gene tree topology and branch lengths or coalescent times). Let $G = \{ G_i \})$. We assume the loci are independent so that

(1) \begin{equation*}f(X \mid G, \psi)=\prod_{i=1}^{L} f\left(X_{i} \mid G_{i}, \Psi\right)\end{equation*}

where $\psi)$ is a vector of parameters in the mutation/substitution model, and $f(X_i|G_i,\psi))$ is the *phylogenetic likelihood* for locus $i)$, calculated according to the usual pruning algorithm (Felsenstein 1981). The posterior probability of the species tree ($S)$) and the parameters is given by

(2) \begin{equation*}\begin{aligned} f(S, \Theta \mid X)= \frac{1}{f(X)} \int_{\psi} \int_{G} f(S) f(\Theta \mid S) f(\psi) \\ f(G \mid S, \Theta) f(X \mid G, \psi) \mathrm{d} G \mathrm{~d} \psi, \end{aligned}\end{equation*}

where $\Theta = \{ \{\tau_j\}, \{ \theta_j \} \})$ is the set of parameters ($\tau)$s and $\theta)$s) associated with the species tree $S)$. Note that $\theta_j = 4N_j \mu)$, where $N_j)$ is the effective population size of (ancestral or contemporary) species $j)$ and $\mu)$ is the mutation rate per generation, while $\tau_j)$ is the age of node $j)$ in the species tree. Both $\theta_j )$ and $\tau_j)$ are measured by sequence distance or the expected number of mutations per site, as are branch lengths or coalecent times in the gene trees (Yang 2002; Rannala and Yang 2003). The term $f(G|S, \Theta))$ is the MSC density of gene trees (topology and coalescent times) given the species tree $S)$ and parameters $\Theta)$ (Rannala and Yang 2003). We use MCMC to generate a sample from the joint posterior density of the species tree $S)$, parameters $\Theta)$ and $\psi)$, and gene trees $(G))$:

(3) \begin{equation*}f(S, \Theta, G, \psi \mid X) \propto f(S) f(\Theta \mid S) f(\psi) f(G \mid S, \Theta) f(X \mid G, \psi),\end{equation*}

The marginal posterior $f(S,\Theta|X))$ is obtained by simply ignoring the gene trees and substitution parameters $(G, \psi))$ in the MCMC sample. Here we focus on two new MCMC proposals that efficiently propose changes to the species tree topology ($S)$). The moves that do not alter the species tree topology are identical to those described in Rannala and Yang (2003, 2013). The first move, based on the SPR algorithm, is a direct extension of the Nearest-Neighbor Interchange (NNI) algorithm implemented in Yang and Rannala (2014). The second move, based on a node-slider algorithm, changes the topology as well as a node age in the species tree.

### The SPR Algorithm for Updating the Species Tree

Let anc($a)$) be the mother node of node $a)$. We refer to the branch anc($a)$)-$a)$ as branch $a)$. We define clade or subtree $a)$ to include $a)$, all its descendents, and branch $a)$. Nodes on the species tree are represented by capital letters, such as $A)$, and their ages are denoted by $\tau)$s (such as $\tau_A)$). Nodes on gene trees are labeled using small-case letters, and their ages are denoted by $t)$s.

Our SPR move prunes off branch $Y)$-$A)$ (including clade $A)$) and reattaches it to a target branch $C)$, retaining the same age $\tau_Y)$ at reattachment (Fig. 1). Our algorithm does not change species divergence times in the species tree ($\tau)$s) or node ages in the gene trees ($t)$s). We preferentially propose changes to the species tree topology around short (rather than long) internal branches. We sample an internal branch $i)$ (out of $s-2)$ internal branches for a species tree of $s)$ species) according to the following probabilities

(4) \begin{equation*}w_{i} \propto b_{i}^{-\frac{1}{2}},\end{equation*}

where $b_i)$ is the length of the internal branch. The sampled branch is branch $X)$-$Y)$. Node $Y)$ has two daughter branches. We sample one at random and let it be $A)$; the other will be $B)$. We then prune off branch $Y)$-$A)$ (including clade $A)$) and reattach it to branch $C)$ in the species tree. Let $Z)$ be the most recent common ancestor of $A)$ and $C)$, with age $\tau_Z)$. The move affects species on the path $A)$-$Z)$-$C)$. For the SPR move illustrated in Figure 1, $Y)$ is species $AB)$, $X)$ is $ABD)$, and $Z)$ is $ABCD)$.

Among the feasible target branches of the species tree for reattachment, we sample one using a probability distribution that favors small changes to the species tree topology. A feasible target branch is a branch that remains after branch $Y)$-$A)$ is pruned off (exclusive of branch $B)$) and that covers the age $\tau_Y)$ (see Fig. 1). In choosing a target branch, we use probabilities

(5) \begin{equation*}v_{i} \propto 1 / c_{i},\end{equation*}

where $c_i)$ is the number of nodes on the path $A)$-$Z)$-$C_i)$ for potential target branch $C_i)$. The minimum for $c_i)$ is 4, in which case node $Z)$ coincides with node $X)$, and the SPR move reduces to the NNI move (Yang and Rannala 2014). Our proposal using Equation (5) thus favours small changes to the species tree topology.

The move affects nodes on the gene trees that have age $\tau_Y \lt t \lt \tau_Z)$. A *moved* node (marked with $\bullet)$ in Fig. 1) lies in species $AB)$ ($Y)$) or another ancestral species on the path from $Y)$ to $Z)$ (excluding $Z)$ itself) and has exactly one daughter node with descendants in $A)$ only. The other daughter node has descendants in one or more non-$A)$ descendent populations as well. The moved node (and the descendant clade) is pruned and regrafted to a randomly chosen contemporary branch of the gene tree residing in a species on the path from $C)$ to $Z)$. In addition, four other kinds of *affected* nodes have their population IDs changed. Any node marked with $\bigcirc)$ or $\bigtriangleup)$ has descendents in species $A)$ only and changes its population ID from $AB)$ ($Y)$) to $AC)$. Any node marked with $\Diamond)$ is in species $C)$ with age between $\tau_Y)$ and $\tau_{anc(C)})$ and changes its population ID from $C)$ to $AC)$. Any node marked with $\square)$ is in species $AB)$ with both daughter nodes having descendants in species $B)$, and changes its population ID from $AB)$ to $B)$. The proposal ratio incurred by the move can easily be derived using a procedure similar to that used for the NNI move (Yang and Rannala 2014).

### Nodeslider Algorithm for Updating the Species Tree

#### Overview of the algorithm.

**Figure 2.**
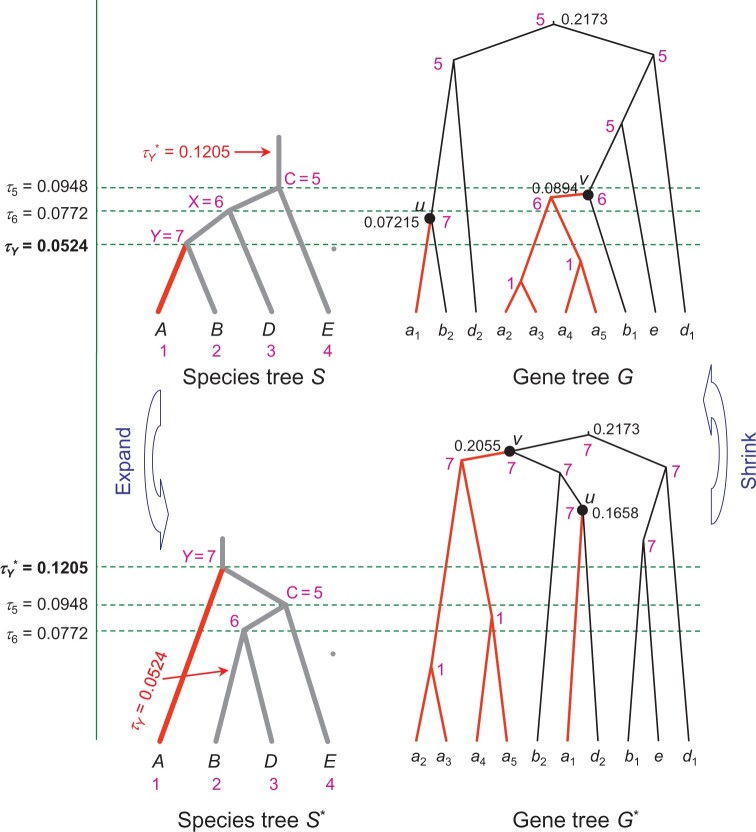
The nodeslider/Expand move (top to bottom) prunes off branch $Y)$-$A)$ on the species tree $S)$ (including clade $A)$), generates a new age for node $Y)$, with $\tau_Y^* \gt \tau_X \gt \tau_Y)$ [Equation (6)], rescales the node ages inside clade $A)$ by $\tau^*_Y/\tau_Y)$, and reattaches branch $Y)$-$A)$ back to the species tree at the ancestral branch $C)$ at age $\tau_Y^*)$ (indicated by the arrow). Affected nodes in the gene tree ($u)$ and $v)$, marked by $\bullet)$) are pruned and regrafted, with node ages inside the clades scaled by $\tau^*_Y/\tau_Y)$. In the reverse Shrink step (from bottom to top), branch $Y)$-$A)$ in $S^*)$ is pruned off and reattached to a descendent branch ($B)$) of the sibling node ($C)$), at the new age $\tau_Y)$, generated from Equation (7). Numeral labels on the interior nodes in the gene trees are the population IDs.

The nodeslider move prunes off branch $Y)$-$A)$ (including clade $A)$) in the species tree, changes $\tau_Y)$ and rescales the ages inside clade $A)$ proportionally, and then reattaches the branch (and clade $A)$) to a target branch in the remaining species tree. This proposal consists of a pair of opposite steps, referred to as the “Expand” and “Shrink” steps (Fig. 2). In the Expand step (toward the root), $\tau_Y)$ increases, and the target branch is ancestral to node $Y)$. In the Shrink step (toward the tips), $\tau_Y)$ decreases, and the target branch is a descendent of the sibling node of $A)$. Thus the move slides node $Y)$ and the attached clade $A)$ either toward the root, with the node ages in clade $A)$ expanded (the Expand step), or to a descendent branch of the sibling species of $A)$, with the node ages in clade $A)$ shrunk (the Shrink step). Figure 2 (from top to bottom) illustrates the changes to the species tree ($S \rightarrow S^*)$) and to an example gene tree ($G \rightarrow G^*)$) in the Expand step. The reverse changes from bottom to top ($S^* \rightarrow S)$ and $G^* \rightarrow G)$) constitute the Shrink step. Note that the sibling and target branches are reversed in the two steps: in the Expand step, $B)$ is the sibling node of $A)$, and $C)$ is the target branch for reattachment, while in the Shrink step, $C)$ is the sibling node and $B)$ is the target branch.

#### Changes to the species tree.

We describe the changes to the species tree first. A uniform random variable $U)$ on $(0,1))$ is generated to decide whether to expand (if $U \geq 0.5)$) or to shrink (if $U \lt 0.5)$).

In the Expand step (from $S)$ to $S^*)$ in Fig. 2), we use Equation (4) to sample an internal branch (out of $s - 2)$) on the species tree and let it be $X)$-$Y)$. Node $Y)$ has two daughter nodes. We sample one at random and let it be $A)$; the other will be $B)$. We then propose a new age $\tau_Y^*)$ for node $Y)$ using an exponential density,

(6) \begin{equation*}f_{+}\left(\tau_{Y}^{*} \mid \tau_{X}\right)=\left(\frac{1}{0.1 \tau_{X}}\right) \mathrm{e}^{-\frac{1}{0.1+\mathrm{x}}\left(\tau_{Y}^{*}-\tau_{X}\right)}, \quad \tau_{\mathrm{X}}\lt \tau_{Y}^{*}\lt \infty . \end{equation*}

In other words, the excess $\tau_Y^* - \tau_X)$ has mean $0.1\tau_X)$. The value $0.1)$ is the “Expand ratio” and is adjustable; we suspect small values close to zero are preferable. We prune off branch $Y)$-$A)$ (including clade $A)$), rescale the ages of all daughter nodes of $A)$ by the factor $\tau_Y^* / \tau_Y)$, and then re-attach the branch to the remaining species tree at age $\tau_Y^*)$. There will be only one ancestral branch (called $C)$) which covers the new age $\tau_Y^*)$. If this is the root, node $Y)$ will become the new root (as in Fig. 2).

The Shrink step is illustrated as the changes from bottom to top in Figure 2. We use Equation (4) to sample an internal branch on the species tree ($S^*)$) and let it be $Y)$-$C)$. The other daughter of node $Y)$ will be $A)$ (i.e., $C)$ is the sibling of $A)$). We prune off branch $Y)$-$A)$ (including clade $A)$), propose a new age $\tau_Y)$ for node $Y)$, rescale all node ages inside clade $A)$ by $\tau_Y/\tau_Y^*)$, and reattach branch $Y)$-$A)$ to a branch ($B)$) that is a descendent of the sibling node ($C)$). Let $G(\tau_Y))$ be the number of descendent branches of $C)$ that exist at time point $\tau_Y)$; in the example of Figure 2, $G(\tau_Y) = 3)$ (for branches $B)$, $D)$, and $E)$). One of them is sampled at random to be the target branch ($B)$). The new age $\tau_Y)$ is proposed using a power density

(7) \begin{equation*}f_{-}\left(\tau_{Y} \mid \tau_{C}, \lambda\right)=\frac{\lambda}{\tau_{C}}\left(\frac{\tau_{Y}}{\tau_{C}}\right)^{\lambda-1}, 0\lt \tau_{Y}\lt \tau_{C}.\end{equation*}

To simulate from the power density we use the inverse transformation method. Generate a uniform random variable $u \sim U(0, 1))$ and set

(8) \begin{equation*}\tau_{Y}=\tau_{C} \times u^{1 / \lambda}.\end{equation*}

Note that Equation (7) becomes the uniform density on $(0, \tau_C))$ if $\lambda = 1)$. We choose $\lambda=\log(0.1)/\log(0.9) = 21.85)$ so that 90% of the density is within 10% of $\tau_C)$ (with $\tau_Y \gt 0.9 \tau_C)$). Here the value 10% is called the “Shrink ratio.” We favor small values like 0.1 so that the new age $\tau_Y)$, smaller than $\tau_C)$, tends to be close to it.

We now consider the factor in the acceptance ratio incurred by changes to the species tree. For the Expand step, this is given as

(9) \begin{equation*}\begin{gathered} R_{\text {Expand }}=\left[\frac{w_{C}^{*} \times 1 \times \frac{1}{G\left(\tau_{Y}\right)} \times f_{-}\left(\tau_{Y} \mid \tau_{C}, \lambda\right)}{w_{Y} \times 0.5 \times 1 \times f_{+}\left(\tau_{Y}^{*} \mid \tau_{X}\right)}\right] \times\left(\frac{\tau_{Y}^{*}}{\tau_{Y}}\right)^{m} \\ \times \frac{g\left(\tau_{0}^{*}\right)}{g\left(\tau_{0}\right)} \times\left(\frac{\tau_{0}^{*}}{\tau_{0}}\right)^{-(s-2)}, \end{gathered}\end{equation*}

where $m)$ is the number of node ages inside clade $A)$ that are rescaled, $g(\cdot))$ is the gamma prior density for the root age $\tau_0)$, and $s - 2)$ is the number of nonroot interior nodes on the species tree. The denominator in the square brackets is for the Expand step, and is the probability ($w_Y)$) of sampling branch $X)$-$Y)$ in $S)$ [Equation (4)], times the probability (0.5) of sampling the daughter $A)$ of node $Y)$, times the probability (1) of choosing target branch ($C)$), times the probability density for the new age $\tau_Y^*)$ [Equation (6)]. The numerator in the square brackets is for the reverse Shrink step (from $S^*)$ to $S)$) and reads as follows: we sample branch $Y)$-$C)$ in $S^*)$ with probability $w_C^*)$, choose node $A)$ as the sibling of $C)$ with probability 1, choose the target branch ($B)$) at age $\tau_Y)$ with probability $1/G(\tau_Y))$, whereas the new age $\tau_Y)$ is generated from Equation (7). The factor $( \frac{\tau_Y^*}{\tau_Y} )^m)$ is due to rescaling $m)$ node ages (see Yang 2014 pp. 225–256). Furthermore, if the move changes the root age ($\tau_0)$) on the species tree, the prior on the node ages in the species tree (the $\tau)$s) has to be considered, which explains the terms involving $\tau_0)$ in Equation (9) (see Yang and Rannala 2010, Equation (2)). Finally, for the reverse Shrink step, the factor in the acceptance ratio is $R_{\textrm{Shrink}} = 1/R_{\textrm{Expand}})$.

#### Changes to the gene trees.

The gene trees are modified to avoid conflicts with the newly proposed species tree, similarly to the SPR algorithm. Some nodes are pruned off the gene tree and regrafted back and some nodes have their population IDs changed due to the disappearance and appearance of populations. We scan the gene tree at each locus to identify the moved nodes. A moved node (marked with $\bullet)$ in Fig. 2) has exactly one daughter node with descendents in $A)$ only. We prune off each moved node (and its $A)$ descendents), rescale the node ages inside the subtree by the scale factor ($\tau_Y^* / \tau_Y)$ for the Expand step) and re-graft the node back to a randomly-chosen branch that exists at the new time $t^* = t \times \frac{\tau_Y^*}{\tau_Y})$. Note that target branches for reattachment must be in a population that is either node $Y)$ or its ancestor in the new species tree ($S^*)$ for the Expand step or $S)$ for the Shrink step, Fig. 2). There may be multiple target branches for reattachment, from which one is chosen at random. For example, in the Shrink move of Figure 2, the new age ($t = 0.07215)$) for the affected node $u)$ will be in population $Y)$ ($AB)$) in the new gene tree $G)$, and two branches ($b_1)$ and $b_2)$) exist in that population and are feasible targets for reattaching the subtree (or branch $u)$-$a_1)$). Similarly affected node $v)$ will be in population $X)$ in $G)$, and four branches ($b_1)$, $b_2)$, $d_1)$, and $d_2)$) are feasible targets for reattaching the subtree.

At every locus, there may be multiple moved nodes and thus multiple subtree pruning and regrafting operations on the gene trees. These are conducted in a disciplined manner, as follows. We prune off all moved nodes (and the subtrees of pure-$A)$ descendents, highlighted in red in Fig. 2), and “lay them on the ground.” For each moved node we then determine the new age after the scaling, sample the target branch for reattachment and mark the reattachment point. The remaining part of the gene tree after pruning off all moved nodes (black branches in the gene trees of Fig. 2), called the skeleton, is not changed except that gene tree nodes in population $Y)$ or in the target population (e.g., $C)$ in the Expand step) may have their population IDs changed. In short, we prune off the red subtrees and reattach them to the black branches on the skeleton (Fig. 2).

The order of pruning and reattachment of the affected nodes is thus inconsequential. In this way, we do not allow regrafting of one pruned branch onto another pruned branch, but it may be possible for multiple pruned subtrees to be reattached to the same branch on the skeleton (at different time points). It is also possible for a pruned branch to be regrafted to the same branch on the skeleton, so that the operation may change the node ages without changing the gene tree topology.

If all sequences at a locus are from populations inside clade $A)$ on the species tree, all node ages on the gene tree are rescaled (in the same way as the moved node), while their population IDs remain unchanged. This rescaling is necessary as otherwise the gene tree may be in conflict with the proposed new species tree.

The changes to the gene trees will incur a factor in the acceptance ratio, because the following components may not be the same in the forward and reverse moves: the number of target branches for reattaching each moved node, the probability density of the gene tree given the species tree topology and parameters ($\tau)$s and $\theta)$s in the MSC density), the rescaling of gene-tree node ages, as well as the probability of the sequence alignment given the gene tree at each locus (the phylogenetic likelihood).

#### The case of three species.

**Figure 3.**
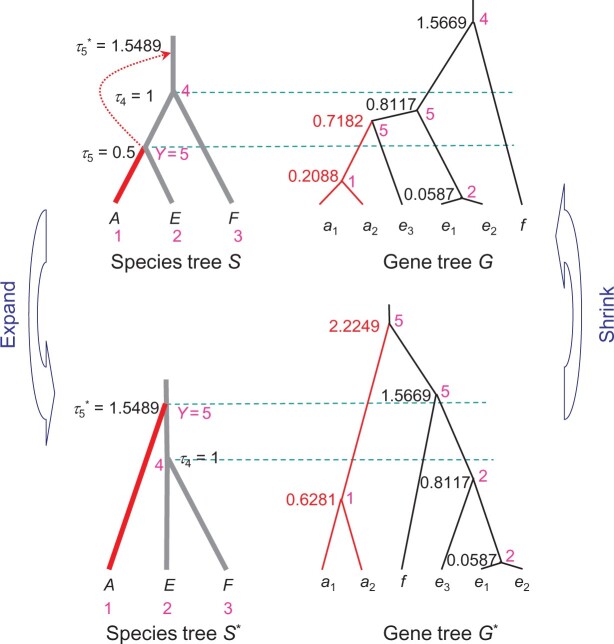
The nodeslider move for three species is a variant of the NNI rearrangement for rooted trees, and changes both the species tree topology and a species divergence time ($\tau)$), and always changes the root of the species tree. The move prunes off branch $Y)$-$A)$ on the species tree, changes $\tau_Y)$, and reattaches the branch to target branch $C)$ on the species tree. In the Expand step, the target branch in the species tree $S)$ is $C = 4)$ (the root), whereas in the Split step, the target branch in the species tree $S^*)$ is $C = 2)$, a descendent of the sibling branch $B = 4)$.

In the case of only three species, the nodeslider move reduces to a variant of the general NNI algorithm for rooted trees (Yang 2014, p. 293), although it differs from the NNI algorithm implemented by Yang and Rannala (2014) or the SPR move described above. The move changes both the species tree topology and a species divergence time ($\tau)$), and always changes the root of the species tree (Fig. 3). In the Expand step (Fig. 3, $S \rightarrow S^*)$), branch $Y)$-$A)$ is pruned off, the age $\tau_Y)$ is increased to $\tau^*_Y \gt \tau_X)$, and the branch is reattached to the species tree, with node $Y)$ becoming the new root. The reverse Shrink step (Fig. 3, $S^* \rightarrow S)$) slides the root of the species tree towards the tips so that the younger interior node becomes the new root.

### Validation of the Theory and Implementation

The new SPR and nodeslider moves are implemented in BPP. Our algorithms are complex and extensive testing has been conducted to confirm the correctness of the theory and the implementation. Because our new moves do not affect the calculation of the phylogenetic likelihood our tests have focused mainly on generating the prior for the species trees and parameters of the MSC model ($\theta)$s and $\tau)$s) via MCMC when the sequence likelihood is fixed at 1. Note that each of the three moves to change the species tree topology that we have implemented, including the NNI of Yang and Rannala (2014) and the SPR and nodeslider moves of this paper, is sufficient to allow the MCMC to traverse the whole space of the species trees. In BPP, we use SPR (which includes NNI as a special case) and nodeslider moves with pre-assigned probabilities (such as 0.6 for SPR and 0.4 for nodeslider). We confirmed that the SPR and nodeslider algorithms, used either alone or in combination, sampled the species trees correctly according to the prior, which is analytically available for four different priors described by Yang and Rannala (2014) and Yang (2015) for the cases of 3, 4, and 5 species.

### Summary of the Posterior

The BPP program generates an MCMC sample from the posterior probability distribution of species trees and the posterior distribution of parameters ($\tau)$s and $\theta)$s) given each species tree. Here we focus on summaries of the species trees. The species tree with the highest posterior probability, called the maximum *a posteriori* (MAP) species tree, is the best point estimate. The MCMC sample can also be used to calculate the support values for clades on the MAP tree. The program also generates posterior probabilities for individual clades as well as the majority-rule consensus tree, with support values. The posterior of model parameters ($\theta)$s and $\tau)$s) on the MAP tree can be generated by using the subset of the MCMC sample in which the species tree is the MAP tree. However, if the model parameters are of interest, one can run the program a second time with the species tree fixed at the MAP tree (analysis A00, Yang 2015). This approach is used to generate the posterior distribution of parameters on the MAP tree in our analysis of the empirical datasets; see Figures 6 and 8.

### Marginal Likelihood Calculation for Fixed Species Trees

Alternative to the transmodel MCMC algorithms we implemented (NNI, SPR, and nodeslider), the posterior probabilities of species trees can easily be calculated if the marginal likelihood under the MSC given the species tree is available:

(10) \begin{equation*}f(X \mid S)=\int_{\Theta} \int_{\psi} \int_{G} f(\Theta \mid S) f(\psi) f(G \mid S, \Theta) f(X \mid G, \psi) \mathrm{d} G \mathrm{~d} \psi \mathrm{d} \Theta.\end{equation*}

As $\frac{f(S_1|X)}{f(S_2|X)} = \frac{f(S_1)}{f(S_2)} \times \frac{f(X|S_1)}{f(X|S_2)})$ for any two alternative species trees $S_1)$ and $S_2)$, the posterior probabilities for rooted species trees are proportional to their marginal likelihood values under the uniform prior on species trees (with $f(S_1) = f(S_2))$, Prior 1 in BPP, Yang and Rannala 2014), while under the uniform prior on labeled histories (Prior 0, Yang and Rannala 2014), the posterior is proportional to the product of the marginal likelihood and the number of compatible labeled histories. Note that the ratio of marginal likelihood values, $\frac{f(X|S_1)}{f(X|S_2)})$, is the Bayes factor.

Here we implement the path-sampling or thermodynamic integration approach to marginal likelihood calculation under the MSC, with the species tree fixed (Gelman and Meng 1998; Lartillot and Philippe 2006). For a simple likelihood model with parameters $\phi)$ and data $x)$, the path-sampling method makes use of the so-called power posterior, defined as

(11) \begin{equation*}f_{\beta}(\phi \mid x) \propto f(\phi) f(x \mid \phi)^{\beta}, 0\lt \beta\lt1,\end{equation*}

which becomes the prior if $\beta = 0)$ or the posterior if $\beta = 1)$, so that different values of $\beta)$ form a path from the prior to the posterior. The logarithm of the marginal likelihood, $f(x) = \int_\phi f(\phi) f(x|\phi) \, \textrm{d}\phi )$, is then given by

(12) \begin{equation*}\log f(x)=\int_{0}^{1} \mathcal{E}_{\beta}\{\log f(x \mid \phi)\} \mathrm{d} \beta,\end{equation*}

where the expectation is taken over the power posterior $f_\beta(\phi | x) )$. We run multiple MCMC algorithms to sample from the power posterior for different values of $\beta)$ to approximate the expectation of the log likelihood, $\mathcal{E}_\beta \{ \log f(x|\phi) \})$, by the MCMC average, and then use numerical integration to calculate the integral of Equation (12).

In our problem, the likelihood function for the species tree and parameters, $f(X|S, \Theta))$, averages over the gene tree topologies and branch lengths (coalescent times), and is not directly calculable. Instead we treat the latent variables (i.e., the gene tree topologies and coalescent times) as parameters, and define the power posterior as

(13) \begin{equation*}\begin{aligned} f_{\beta}(\Theta, \psi, G \mid X, S) \propto[f (\Theta \mid S) f(\psi) f(G \mid S, \Theta)] \\ \times f(X \mid G, \psi)^{\beta}, 0\lt \beta\lt1,\end{aligned}\end{equation*}

so that $f(\Theta|S) f(\psi) f(G|S, \Theta))$ becomes the joint prior while $f(X|G,\psi))$ is the likelihood. The general procedure of Equation (12) then applies, with

(14) \begin{equation*}\log f(X \mid S)=\int_{0}^{1} \mathcal{E}_{\beta}\{\log f(X \mid G, \psi)\} \mathrm{d}, \beta\end{equation*}

where the expectation $\mathcal{E}_\beta )$ in the integrand is over the power posterior of Equation (13). Calculation based on Equation (14) then shares all the statistical properties of calculation based on Equation (12), such as consistency and unbiasedness (Gelman and Meng 1998). This algorithm has the same structure as the algorithms for calculating the Bayes factors for two substitution models, averaging over different phylogenetic trees, discussed by Wu et al. (2014). Those authors provided a mathematical proof that such algorithms are statistically consistent even though the phylogeny varies in the MCMC. The argument above treating latent variables (gene trees or phylogenies) as parameters appears to be simpler.

We use Gaussian quadrature to approximate the one-dimensional integral over $\beta$, using $K = 16$ points in the Gauss-Legendre rule. The $\beta$ values are given as $\beta_k = \frac{1}{2}(x_k + 1)$, for $k = 1, ..., K$, where $x_k$, with $-1 \lt x_k \lt 1$, are the Gauss–Legendre points. This samples $\beta$ values more densely close to 0 and 1, in comparison with the trapezoid or Simpson methods which use equally spaced points. For each $\beta_k$, we run an MCMC algorithm to generate a sample from the power posterior distribution, and then calculate the average of $\log f(X|G,\psi)$ over the MCMC sample as an approximate to the expectation $\mathcal{E}_{\beta_k} \{ \log f(X|G,\psi) \} $. The integral or the log marginal likelihood is then approximated by

(15) \begin{equation*}\log f(X \mid S) \approx \frac{1}{2} \sum_{k=1}^{K} w_{k} \times \mathcal{E}_{\beta_{k}}\{\log f(X \mid G, \psi)\},\end{equation*}

where $w_k$ are the Gauss–Legendre weights.

Two factors may affect the accuracy of the approximation. First the integrand or the expected log likelihood $\mathcal{E}_{\beta} \{ \log f(X|G,\psi) \} $ is not calculated exactly but approximated by the average over the MCMC sample from the power posterior. Second, the number of quadrature points $K$ is finite. The first factor appears to be much more important. In particular, for small values of $\beta$ and for large datasets, the power posterior may differ substantially from the likelihood. As a result the log likelihood is very small for most values of $(G, \psi, \Theta)$ sampled from the power posterior, but is huge occasionally, making it difficult to estimate its average. Note that the posterior probability ratio between two species trees is related to the difference in log marginal likelihood ($\Delta \ell$) by $P_1/P_2 = \mathrm{e}^{\Delta \ell}$. As $\mathrm{e}^{\Delta \ell + \delta} \approx \mathrm{e}^{\Delta \ell}(1 + \delta)$, where $\delta$ is the small error, we need the log marginal likelihood difference or the expected log likelihood to be accurate at the 1st (or 2nd) decimal point for the relative error in the posterior probability to be 10% (or 1%). This level of precision may require very long chains to simulate the power posterior. In contrast, the second factor may not be important and $K = 16$ may be large enough as in previous applications of the quadrature method, with exact calculation of the integrand, use of 8 or 16 points provided excellent approximations to one-dimensional integrals (Zhu and Yang 2012; Yang 2014 p. 206–209).

Thus although the challenge of the transmodel MCMC algorithms (the NNI, SPR, and nodeslider) lies in the difficulty of moving from one species tree to another, the challenge of the path-sampling approach to marginal likelihood calculation appears to lie mainly in the reliable estimation of the expectation of the log likelihood over the power posterior. In addition the algorithm of Equation (15) requires $K$ MCMC runs. The algorithm may be useful for evaluating a few alternative species trees.

## Results

### Simulation to Evaluate the Statistical Performance of the Method

**Figure 4.**
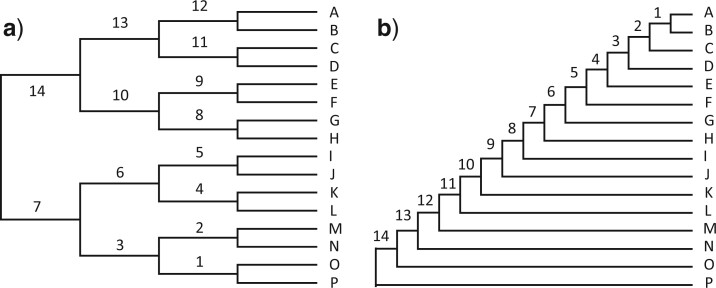
Symmetrical a) and asymmetrical b) species trees used in computer simulation to evaluate the performance of the BPP program. The branches are drawn to represent their lengths ($\tau$s) and the 14 nodes are labeled in each tree.

Simulations were used to examine the influence on the posterior probabilities of species trees of the number of loci, the mutation rate (sequence divergence level), and the prior on topology. We simulated data under the MSC using either a completely symmetrical or asymmetrical tree of 16 species, with two sequences sampled per species per locus (see Fig. 4). For simplicity, we assumed equal $\theta$s among ancestral and contemporary species with either $\theta=0.001$ (low mutation rate) or $\theta=0.01$ (high mutation rate). We set all internal branch lengths equal to $\theta$, so that $\tau_i - \tau_j = \theta$ where node $i$ is the mother of node $j$. Thus, the height of the root was $\tau_0 = 15 \times \theta$ for the asymmetrical tree and $\tau_0 = 4 \times \theta$ for the symmetrical tree. For each of the $2 \times 2 = 4$ parameter/topology combinations 50 datasets were simulated of either $L=2$ or $L=10$ unlinked loci, each with $n=1000$ sites. Thus, $2 \times 2 \times 2 \times 50 = 400$ datasets were simulated in total. The MCcoal program which is part of the BPP package was used to generate gene trees under the MSC and to simulate sequence alignments on the trees under the JC69 model.

The simulated datasets were analyzed using the BPP 3.2 program with a $G(2, 200)$ prior for $\theta$ when the true $\theta=0.01$ and a $G(2, 2000)$ prior for $\theta$ when the true $\theta=0.001$. Although the prior means match the true values, the gamma distribution with shape parameter 2 is diffuse (uninformative). Similarly, a gamma prior with shape parameter 2 and with mean equal to the true value was assigned to $\tau_0$, the age of the root of the species tree. In other words, the prior on $\tau_0$ was $G(2, 50)$ for datasets simulated under a symmetrical tree with $\theta=0.01$, $G(2, 13.3)$ for the asymmetrical tree with $\theta=0.01$, $G(2, 500)$ for the symmetrical tree with $\theta=0.001$, and $G(2, 133)$ for datasets simulated under an asymmetrical tree with $\theta=0.001$. Two analyses were carried out for each dataset using different priors on the tree topology: a uniform prior on labeled histories (Prior 0, Yang and Rannala 2014) and a uniform prior on rooted trees (Prior 1). Each of the simulated datasets (and prior combinations) was analyzed using two independent MCMC runs with either a good starting species tree (the true species tree) or a poor starting species tree to check for consistency between runs. Thus $400 \times 2 \times 2 = 1600$ MCMC runs were carried out in total. Each MCMC analysis was run for 200,000 iterations, sampling every second iteration and discarding the first 50,000 iterations as burn-in.

To examine the statistical performance of the method we calculated the proportion of datasets (among 50 replicate simulations) in which each of the 14 nodes in the true species tree is found in the consensus tree; note that a node of the true tree is in the consensus tree if its posterior probability is $\gt 0.5$. This is a measure of power. We also examined the empirical coverage of the $95\%$ and $99\%$ Credible Set of Trees (CST). Coverage is defined as the proportion of credible sets that contain the true tree. The results are summarized in Table 1. The method performs very well in identifying the true clades, even with only 2 loci. With the exception of nodes 12 to 14 at the base of the tree (see Fig. 4) all nodes of the true tree are present in the consensus tree with frequencies of 0.76 or greater. The empirical coverage of the credible set of trees provides a measure of the accuracy of the method. The accuracy is very high, with the true tree contained in both the 95% and 99 % credible sets in all cases (with the realized coverage to be 100%) except two: (i) trees inferred using Prior 0 from data simulated on an asymmetrical tree with 2 loci and with $\theta=0.001$ — the coverage is 0.92 for the $95\%$ and $99\%$ CSTs; and (ii) trees inferred using Prior 1 from the data simulated on a symmetrical tree with 2 loci and with $\theta=0.001$ — the coverage is 0.98 for the $95\%$ CST and 1.0 for the $99\%$ CST. In other words, in all but one case the coverage is greater than the nominal value of either $95\%$ or $99\%$.

**Table 1.** Summary of results for simulation analyses

	Number of loci: 2								Number of loci: 10							
	Symmetrical tree				Asymmetrical tree				Symmetrical tree				Asymmetrical tree			
	$\theta=0.01$		$\theta=0.001$		$\theta=0.01$		$\theta=0.001$		$\theta=0.01$		$\theta=0.001$		$\theta=0.01$		$\theta=0.001$	
Prior	LH	T	LH	T	LH	T	LH	T	LH	T	LH	T	LH	T	LH	T
Node	Proportion of datasets with true node present in consensus tree															
1	0.98	0.98	0.80	0.76	0.98	1.0	0.88	0.86	1.0	1.0	1.0	1.0	1.0	1.0	1.0	1.0
2	1.0	0.98	0.90	0.84	0.94	0.96	0.88	0.90	1.0	1.0	1.0	0.98	1.0	1.0	1.0	1.0
3	1.0	1.0	0.88	0.74	0.98	1.0	0.84	0.86	1.0	1.0	1.0	1.0	1.0	1.0	1.0	1.0
4	0.98	0.94	0.92	0.92	0.96	1.0	0.82	0.84	1.0	1.0	1.0	1.0	1.0	1.0	1.0	1.0
5	0.96	0.92	0.90	0.88	0.94	0.98	0.76	0.80	1.0	1.0	1.0	1.0	1.0	1.0	1.0	1.0
6	1.0	1.0	0.90	0.80	0.96	0.98	0.78	0.84	1.0	1.0	1.0	1.0	1.0	1.0	1.0	1.0
7	0.96	0.96	0.76	0.64	0.96	0.98	0.76	0.80	1.0	1.0	0.98	0.98	1.0	1.0	1.0	1.0
8	1.0	0.98	0.88	0.82	0.96	1.0	0.84	0.86	1.0	1.0	1.0	1.0	1.0	1.0	0.98	1.0
9	0.98	0.98	0.90	0.90	0.94	0.98	0.86	0.88	1.0	1.0	1.0	1.0	1.0	1.0	1.0	1.0
10	0.96	0.96	0.80	0.78	0.98	1.0	0.86	0.90	1.0	1.0	1.0	1.0	1.0	1.0	1.0	1.0
11	0.98	0.94	0.80	0.76	0.96	1.0	0.76	0.80	1.0	1.0	1.0	1.0	1.0	1.0	1.0	1.0
12	0.98	0.96	0.92	0.88	0.90	0.96	0.68	0.80	1.0	1.0	1.0	1.0	1.0	1.0	1.0	1.0
13	0.96	0.96	0.76	0.68	0.86	0.94	0.36	0.64	1.0	1.0	1.0	1.0	1.0	1.0	0.98	1.0
14	0.96	0.94	0.78	0.74	0.66	0.80	0.13	0.46	1.0	1.0	1.0	1.0	0.94	0.98	0.58	0.80
CST	Empirical coverage															
95%	1.0	1.0	1.0	0.98	1.0	1.0	0.92	1.0	1.0	1.0	1.0	1.0	1.0	1.0	1.0	1.0
99%	1.0	1.0	1.0	1.0	1.0	1.0	0.92	1.0	1.0	1.0	1.0	1.0	1.0	1.0	1.0	1.0
CST	Mean number of trees in 99% CST															
	243.5	372.8	4375.7	6297.3	443.9	200.9	6205.8	2711.7	1.1	1.1	15.6	19.1	3.7	3.0	46.9	26.3

*Notes:* The upper matrix shows the proportion of simulated datasets for which each node of the true species tree is present in consensus tree. The empirical coverage of the 95% and 99% credible sets of trees (CSTs) tabulates the proportion of simulated datasets (across 50 simulated datasets for each set of simulation conditions) for which the true tree is contained within the credible set. The mean number of trees in the CST is the average number of trees in the 99% CST (averaging across 50 simulated datasets for each set of simulation conditions). Each dataset is analyzed using 2 species tree priors: the uniform prior for labeled histories (LH) and the uniform prior for rooted trees (T). Node numbers are shown in Figure 4.

**Figure 5.**
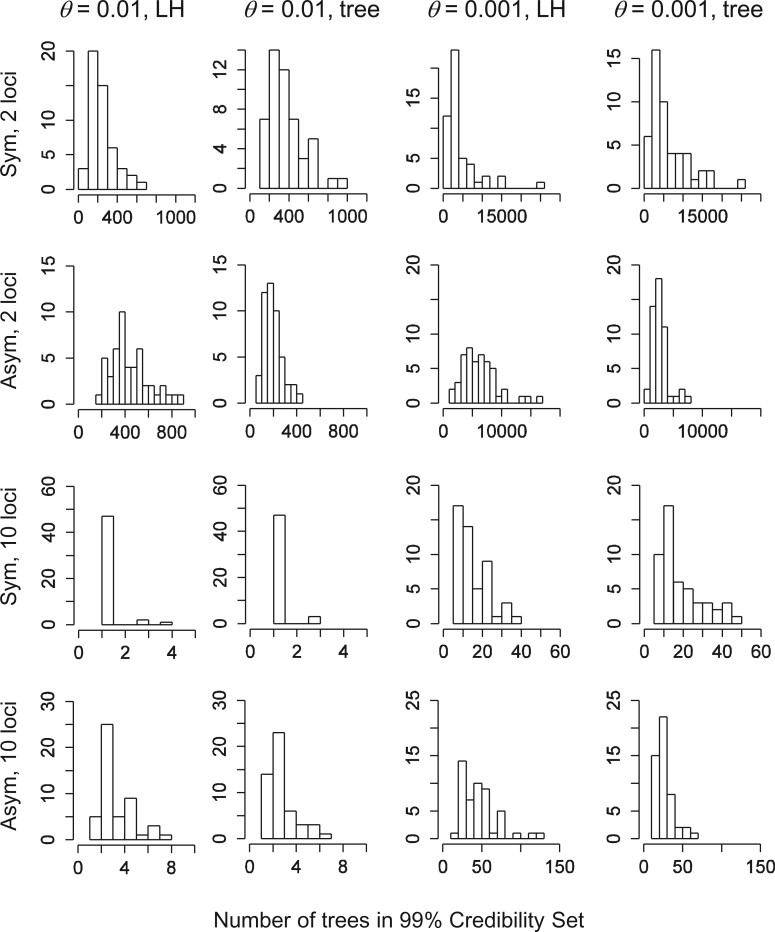
Histograms of the number of species trees in the 99% credible set from analyses of 50 simulated datasets for each of 8 combinations of simulation conditions and two different species tree priors: Prior 0 (LH) which assigns equal probabilities to labeled histories (columns 1 and 3), and prior 1 (tree) which assigns equal probabilities to rooted species trees (columns 2 and 4). The upper two rows show results for two loci and and the lower two rows results for 10 loci. Rows 1 and 3 are results for data simulated on symmetrical (Sym) trees and rows 2 and 4 for asymmetrical (Asym) trees. The two columns to the left are simulations using $\theta=0.01$ and the two columns on the right are those using $\theta=0.001$.

The mean number of trees contained in the 99% CST provides a measure of the precision of the estimator of species tree topology (Fig. 5 and Table 1). The mean number of trees ranged from a minimum of $1.1$ (for 10 loci, $\theta=0.01$ and a symmetrical true tree with Prior 0) to a maximum of $6297.3$ (for 2 loci, $\theta=0.001$ and a symmetrical true tree with Prior 1). The prior on species trees can have a large effect on the precision of the method (Fig. 5 and Table 1). Prior 0 favors symmetrical trees whereas Prior 1 favors asymmetrical trees, and when the prior favors the shape of the true tree, the estimates are more precise with a smaller CST. When the true tree is symmetrical (Fig. 5, rows 1 and 3), there are fewer trees in the 99% CST under Prior 0 than under Prior 1, whereas the opposite is true when the true tree is asymmetrical (Fig. 5, rows 2 and 4). The impact of the prior is less important when the number of loci increases from 2 to 10 and is negligible for the informative data simulated using $\theta=0.01$ (see Table 1 and Fig. 5).

### Analysis of the Rattlesnake Data

We analyze here the dataset of 18 nuclear loci from six subspecies of *Sistrurus* rattlesnakes, generated and analyzed by Kubatko et al. (2011). Rattlesnakes are venomous snakes of the New World, with species falling into two genera: *Crotalus* which contains more than 20 species and *Sistrurus* which contains three named species: *catenatus, miliarius*, and *ravus*. However, mtDNA suggests that *ravus* in fact belongs to the genus *Crotalus* (Murphy et al. 2002; Parkinson et al. 2002). The data analyzed here are from *S. catenatus* and *S. miliarius* only. Within each of these two species, three subspecies are formally described on the basis of morphological variation in scale characters, body size and coloration, and geographic distribution. The three *S. catenatus* subspecies are *S. c. catenatus* (C), *S. c. tergeminus* (T), and *S. c. edwardsii* (E), whereas the three *S. miliarius* subspecies are *S. m. miliarius* (M), *S. m. barbouri* (B), and *S. m. streckeri* (S). The data also include sequences from two outgroup species: *Agkistrodon contortrix* (Ac) and *A. piscivorus* (Ap). Although the BPP analysis does not require outgroups and those two outgroup species appear quite distant from the ingroup species, we use them as well for easy comparison with the results of Kubatko et al. (2011). We analyze the 18 nuclear loci and the single mitochondrial locus separately, since they have very different characteristics, including different mutation rates and effective population sizes.

#### The nuclear loci.

Among the 18 loci, the number of sequences per locus ranges from 48 to 52, and the sequence length ranges from 194 to 849 (Kubatko et al. 2011, table 2). We use the uniform prior for rooted species trees (Prior 1, Yang and Rannala 2014). For the parameters on the species tree, we use the gamma prior $\theta \sim G(2, 1000)$ with the prior mean 0.002 (2 differences per kb), and $\tau_0 \sim G(1.2, 100)$ with the prior mean for the age of the root to be 0.012. Those parameters of the shape parameter (2 and 1.2) specify diffuse gamma priors, while the means are chosen to be plausible for the data, based on preliminary runs of the A00 analysis (speciesdelimitation = 0, speciestree = 0) under a reasonable tree (Yang 2015). We use 8000 iterations for the burnin, after which we take $2 \times 10^5$ samples, sampling every 4 iterations. We run each analysis twice, with different starting models (species delimitations and/or species trees), to check for consistency between runs and then merge the samples to produce posterior summaries. Each run took about 10 hours on one CPU core. Kubatko et al. (2011) reported running times of $\sim$10 days using *BEAST in previous analyses of those data.

**Table 2.** Summary of results obtained from BPP analysis of the rattlesnake datasets

	28 nuc loci, one rate	28 nuc loci, gamma rate G(2)	ATP (665 bp)
	$\tau_0 \sim G(1.2,100)$	$\tau_0 \sim G(1.2, 100)$	$\tau_0 \sim G(1.5, 10)$
	$\theta_0 \sim G(2, 1000)$	$\theta_0 \sim G(2, 1000)$	$\theta_0 \sim G(2, 1000)$
A00 estimates			
$\quad\tau_0$ (root)	0.0125 (0.0097, 0.0154)	0.0139 (0.0100, 0.0172)	0.145 (0.127, 0.164)
$\quad\tau_1$ (CET-SMB)	0.0042 (0.0033, 0.0052)	0.0044 (0.0034, 0.0054)	0.106 (0.088, 0.124)
$\quad\tau_2$ outgroup	0.0026 (0.0013, 0.0039)	0.0026 (0.0014, 0.0039)	0.062 (0.047, 0.077)
A11 analysis			
$\quad\textrm{ Pr(MS)}$	0.000	0.000	0.968
$\quad\textrm{ Pr(MB)}$	0.020	0.015	0.000
$\quad\textrm{ Pr(ET)}$	0.000	0.000	0.335
$\quadP_4$	0.000	0.000	0.330
$\quadP_5$	0.020	0.015	0.652
$\quadP_6$	0.980	0.985	0.018
A01 analysis			
$\quad\textrm{ (MB)-S}$	0.696	0.608	0.013
$\quad\textrm{ (MS)-B}$	0.232	0.313	0.977
$\quad\textrm{ (BS)-M}$	0.061	0.071	0.011

**Figure 6.**
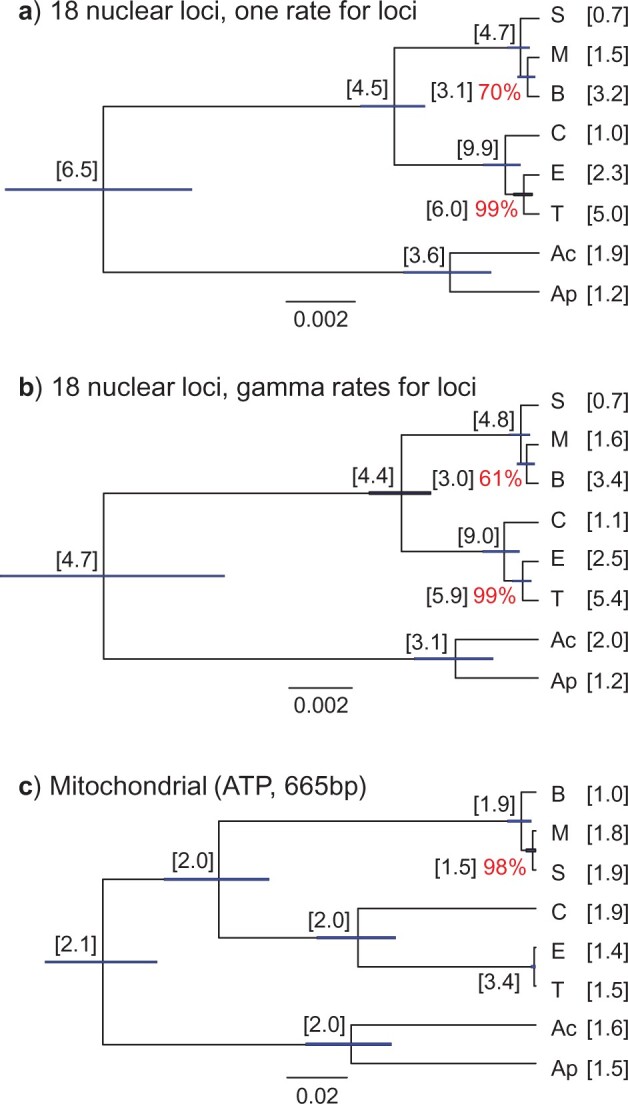
The MAP trees for the six subspecies of *Sistrurus* rattlesnakes and the outgroups in three analyses of the nuclear (18 loci) and mitochondrial datasets. The three *S. catenatus* subspecies are *S. c. catenatus* (C), *S. c. tergeminus* (T), and *S. c. edwardsii* (E), whereas the three *S. miliarius* subspecies are *S. m. miliarius* (M), *S. m. barbouri* (B), and *S. m. streckeri* (S). Posterior probabilities for clades in the species tree in the A01 analysis are shown next to the nodes as percentages (not shown if 100%). The branch lengths are drawn to represent the posterior means of the divergence times ($\tau$s) in the A00 analysis with the phylogeny fixed, whereas the node bars represent the 95% HPD interval. The posterior means of $\theta$s for the extant and extinct species from the A00 analysis are shown next to the nodes in brackets.

We conducted two analyses. In the first, we inferred both the species delimitation and species phylogeny (A11: speciesdelimitation = 1, speciestree = 1). The posterior probability is 98.0% that all the six subspecies are distinct species, with 2% probability that M and B are one species. The best supported phylogeny is shown in Figure 6, and this has posterior probability 69.2%. The next two trees have different relationships for the three subspecies of *S. miliarius* (B, M, and S) from the MAP 3 of Figure 6, with posterior probability 21.8% for (B, (M, S)), and 6.3% for (M, (B, S)). Together the three trees have a cumulative posterior probability of 97.3% and constitute the 97.3% credible set of the species-delimitation and species-tree models.

In the second analysis (A01: speciesdelimitation = 0, speciestree = 1), we treated the 8 species/subspecies as distinct to infer the species tree. As in the first analysis, the top 3 trees differ concerning the relationships among B, M, and S, with posterior probability 71.0% for ((M, B), S) (the MAP 3 of Fig. 6), 22.4% for (B, (M, S), and 6.1% for (M, (B, S)), with the total posterior for all 3 trees to be 99.5%. Because the A01 analysis evaluates a subset of the models considered in the A11 analysis, the posterior probabilities for the shared models in the two analyses should be proportional.

**Figure 7.**
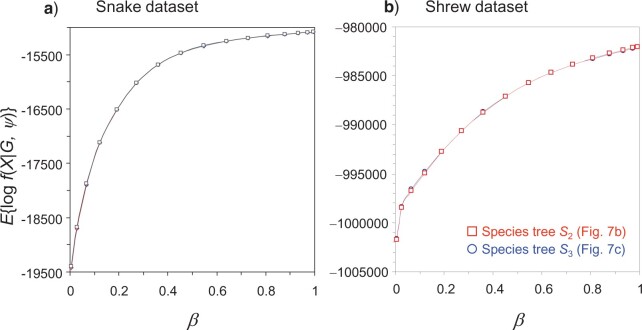
Calculation of the marginal likelihood for fixed species trees. The average log likelihood over the MCMC sample from the power posterior is plotted against the given $\beta$ for each fixed species tree. In a) the 3 alternative species trees for the rattlesnake dataset are compared: MB-S (which is the MAP tree), MS-B, and BS-M (Fig. 6). The three curves are indistinguishable. In b), the 2 curves are for the species trees $S_2$ and $S_3$ of Philippine shrews for the UCE dataset (Fig. 8).

We applied the algorithm of Equation (15) to calculate the log marginal likelihood values for the top three species trees, in comparison with the transmodel MCMC analysis (A01). We use $K = 16$ quadrature points, and run 16 MCMC analyses to generate MCMC samples from the power posteriors. The average log likelihood for given $\beta$ is calculated by averaging over the MCMC sample. Each MCMC run is an A00 analysis. We use 16,000 iterations for the burnin, after which we take $2 \times 10^6$ samples, sampling every 4 iterations. Each run used one core and took 2–4 days. The average log likelihood is plotted in Figure 7a against $\beta$ for each species tree. The log marginal likelihood is then the area under the curve over the interval $\beta \subset (0, 1)$. Equation (15) then gives the log marginal likelihood values as $-15849.54, -15850.57$, and $-15851.80$ for the 3 species trees. With Prior 1, those species trees have the same prior probability, so that their posterior probabilities are proportional to their marginal likelihood values. Thus the posterior probability ratios are $P_1 : P_2 : P_3 = 1 : 0.35 : 0.10$, in comparison with $0.710 : 0.224 : 0.062 = 1 : 0.32 : 0.09$, obtained from the transmodel MCMC results in the A01 analysis above. The two approaches are largely consistent. The discrepancies appear to be due to the inaccuracies in the marginal likelihood calculation, or in the expectation of the log likelihood across the MCMC sample from the power posterior.

As the 18 nuclear loci show considerable rate variation (Kubatko et al. 2011, Table 2), we repeated the analysis using a Gamma-Dirichlet model to account for the mutation rate variation among loci (Burgess and Yang 2008). The gamma parameter in the model is fixed at $\alpha = 2$. This has a small effect on the parameter estimates in the A00 analysis and on the posterior probabilities on the A11 and A01 analyses. The results are summarized in Table 2.

We used this dataset to examine the impact of the priors on parameters in the MSC model ($\theta$s and $\tau$s) on the posterior probabilities of species trees in the A01 analysis (speciesdelimitation = 0, speciestree = 1). We treated the analysis of Table 2 with the priors $\theta \sim G(2, 1000)$ and $\tau_0 \sim G(1.2, 100)$ as the “standard” analysis and changed either the mean or the shape parameter of the gamma prior for either $\theta$ or $\tau$ (table 3). In all those analyses, the MAP species tree and indeed the top three species trees remained the same (table 3). Changes to the prior on $\tau_0$ had virtually no effect on the posterior probabilities of the species trees. In contrast, the $\theta$ prior had considerable impact. When the shape parameter for the $\theta$ prior is fixed, the prior mean had a complex effect, with both small and large $\theta$s leading to reduced support for the MAP species tree. When the prior mean for $\theta$ is fixed, larger shape parameters (or highly concentrated priors) led to increased posterior for the MAP tree. Previously Leaché and Rannala (2011) highlighted the impact of the $\theta$ prior on species tree inference by the Bayesian method BEST (Liu 2008), finding that misspecified priors produced inflated posterior probabilities for species trees.

**Table 3.** The impact of the priors for parameters in the MSC model on posterior probabilities of species trees in the BPP analysis of the rattlesnake dataset (18 nuclear loci)

Species tree (Fig. 6a)	(MB)-S	(MS)-B	(BS)-M
Prior mean for $\theta$, with $\tau_0 \sim G(1.2, 100)$			
$\quad\theta \sim G(2, 10000)$, with mean 0.0002	0.57	0.31	0.10
$\quad\theta \sim G(2, 5000)$, with mean 0.0004$^{\rm a}$	0.42	0.50	0.08
$\quad\theta \sim G(2, 1000)$, with mean 0.002	0.70	0.23	0.06
$\quad\theta \sim G(2, 500)$, with mean 0.004	0.69	0.22	0.08
$\quad\theta \sim G(2, 100)$, with mean 0.02	0.51	0.30	0.20
$\quad\theta \sim G(2, 50)$, with mean 0.04	0.42	0.33	0.24
Prior shape for $\theta$, with $\tau_0 \sim G(1.2, 100)$			
$\quad\theta \sim G(0.5, 250)$, with mean 0.002	0.65	0.27	0.08
$\quad\theta \sim G(1, 500)$, with mean 0.002	0.69	0.24	0.06
$\quad\theta \sim G(2, 1000)$, with mean 0.002	0.70	0.23	0.06
$\quad\theta \sim G(10, 5000)$, with mean 0.002	0.82	0.12	0.05
Prior mean for $\tau$, with $\theta \sim G(2, 1000)$			
$\quad\tau_0 \sim G(1.2, 1000)$, with mean 0.0012	0.71	0.22	0.06
$\quad\tau_0 \sim G(1.2, 100)$, with mean 0.012	0.71	0.22	0.06
$\quad\tau_0 \sim G(1.2, 10)$, with mean 0.12	0.70	0.23	0.06
Prior shape for $\tau$, with $\theta \sim G(2, 1000)$			
$\quad\tau_0 \sim G(0.5, 41.7)$, with mean 0.012	0.71	0.23	0.06
$\quad\tau_0 \sim G(1, 83.3)$, with mean 0.012	0.71	0.22	0.06
$\quad\tau_0 \sim G(2, 166.7)$, with mean 0.012	0.70	0.23	0.06
$\quad\tau_0 \sim G(10, 833.3)$, with mean 0.012	0.70	0.23	0.06

$^{\rm a}$ In the analysis using the priors $\theta \sim G(2, 5000)$ and $\tau_0 \sim G(1.2, 100)$, there is posterior uncertainty concerning the relationships of E, T, and C, as well as uncertainties concerning M, B, and S. The 95% HPD set consists of 7 species trees: (B(MS))-(C(ET)), with posterior 36%, (S(MB))-(C(ET)) with 30%, (B(MS))-(E(CT)) with 10%, (S(MB))-(E(CT)) with 8%, (M(BS))-(C(ET)) with 6%, (B(MS))-(T(CE)) with 5%, and (S(MB))-(T(CE)) with 4%. In all other analyses listed in the table, only the 3 species trees that correspond to the different resolutions of M, B, and C have substantial probabilities.

#### The mitochondrial locus (ATP, 665 bp).

The parameters on the species tree are assigned the following priors: $\theta \sim G(2, 1000)$ with the prior mean 0.002 and $\tau_0 \sim G(1.5, 10)$ with the prior mean 0.15. All other settings are the same as for the analysis of the nuclear loci. The mitochondrial locus favored 5 species, with M and S grouped into one species in all analyses, in contrast to the nuclear loci, which supported the distinct species status of all the 6 subspecies (Table 2). Similarly, the A01 analysis groups M and S together with posterior probability 97.7%. The A00 estimates of species divergence times ($\tau$) are shown in Figure 6c. The branches in the mitochondrial species tree are much longer than for the nuclear loci, indicating that the mitochondrial locus has a much higher mutation rate. As a result, the single mitochondrial locus appears to be at least as informative as the 18 nuclear loci.

The estimated species trees from the nuclear and mitochondrial data have strikingly different shapes. Relative to the root of the tree, the mitochondrial species tree has much older nodes for separation of *S. catenatus* and *S. miliarius*, and for the separation of the the 2 outgroup species: *A. contortrix* and *A. piscivorus*. In the simplistic model of random mating and neutral evolution of both nuclear and mitochondrial loci, the species divergence time parameters ($\tau$s) should be proportional for the nuclear and mitochondrial loci. The ratio of the posterior means of the species divergence times ($\tau$s) between the mitochondrial locus and the nuclear loci is 12 for the root of the species tree, 25 for the common ancestor of *S. catenatus* and *S. miliarius*, and 24 for the divergence of the 2 outgroup species: *A. contortrix* and *A. piscivorus*. If the absolute species divergence times are the same for the nuclear and mitochondrial genomes, those estimates indicate that the mitochondrial mutation rate is 12–25 times as high as the nuclear mutation rate. With such mutation rate differences and if the mitochondrial population size is $\frac{1}{4}$ that for the nuclear loci, we would expect the population size parameters on the species tree ($\theta$s) for the mitochondrial locus to be 3–6 times as large as those for the nuclear loci. Yet, the average of the posterior means for $\theta$s over the populations on the species tree is 0.0019 for the mitochondrial locus and 0.0037 for the nuclear genes, with a ratio of 0.51 (Fig. 6), whereas the average of the ratios is 0.81. Thus the mitochondrial $\theta$s are far smaller than expected from the simple neutral model. In summary, both the fact that the $\tau$ estimates are not proportional between the nuclear and mitochondrial loci and the fact that the $\tau$ and $\theta$ estimates are not proportional suggest that the differences between the nuclear and mitochondrial loci cannot be entirely explained by differences in mutation rates and population sizes alone, and the idealized model does not fit the data. We suggest that extending the mitochondrial locus and sequencing more nuclear loci may be useful for understanding the major factors causing the conflicting signals.

Kubatko et al. (2011) conducted a number of phylogenetic and coalescent-based analyses of the same data. The coalescent-based analyses used the heuristic method STEM (Kubatko et al. 2009) and the Bayesian MCMC method *BEAST (Heled and Drummond 2010). The 18 nuclear loci and the mitochondrial locus were analyzed as one single dataset. The species tree inferred in the *BEAST analysis is the tree shown in Figure 6c, with a posterior probability 0.93 for the M-S grouping. This may be explained by the fact that the mitochondrial locus has a much higher mutation rate so that the signal from the single mitochondrial locus has dominated the analysis when the nuclear and mitochondrial loci are analyzed together. Note that in our BPP analysis, the relationships among B, M, and S are uncertain, and the mitochondrial locus favors the M-S grouping (Fig. 6c and Table 2). Overall our results are largely consistent with those of Kubatko et al. (2011).

### Analysis of Philippine Shrew Datasets

**Figure 8.**
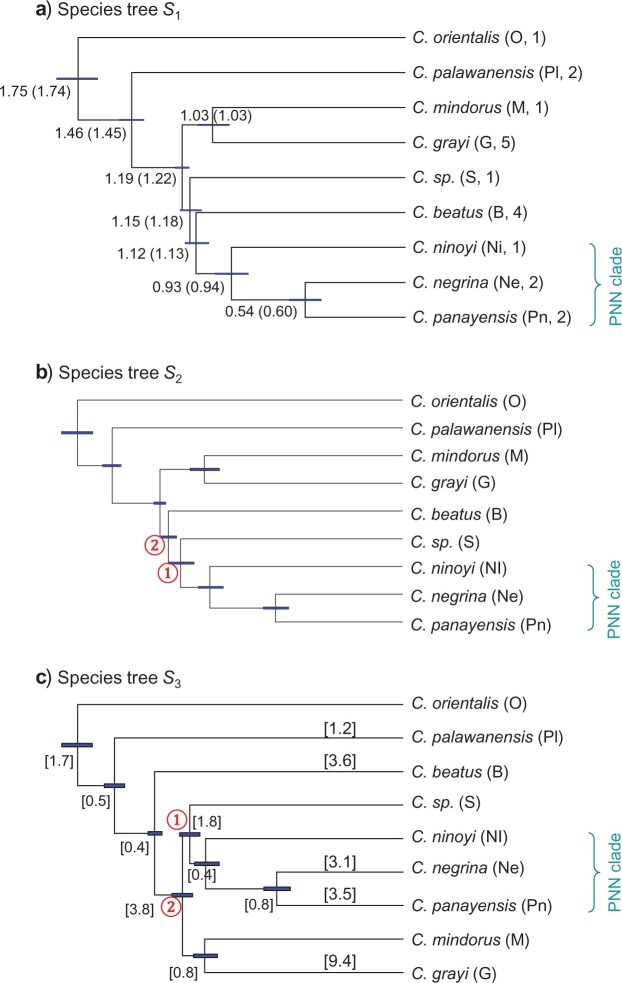
a) The MAP species tree (also the majority-rule consensus species tree) produced in the BPP analysis of the simulated dataset Sim1-Matching of Giarla and Esselstyn (2015). Posterior probabilities for nodes are shown as percentages, whereas those not shown are 100%. Branches are drawn to reflect the posterior means of the node ages ($\tau$s), which are shown next to the nodes (with the true values in parentheses, $\times 1000$), whereas the node bars indicate the 95% highest posterior density (HPD) intervals. The number of sequences per species per locus is in the parentheses after each species name. This tree is also the species tree that Giarla and Esselstyn (2015) inferred from the UCE data and is the true species tree used to generate the three simulated datasets (Sim1, Sim2, and Sim3x). b) and c) Two alternative species trees ($S_2$ and $S_3$) with high posterior probabilities in the BPP analysis of the UCE dataset. $S_3$ appears to be the MAP tree, and the posterior means of $\theta$s for modern and ancestral populations on $S_3$ are shown in square brackets ($\times 1000$). The 95% HPD intervals of node ages in $S_2$ and $S_3$, shown as node bars, are shorter than those in $S_1$, probably because there are 919 loci in the UCE dataset and 500 in Sim1. Note that $S_1$ and $S_2$ differ by an NNI move around node 1, whereas $S_2$ and $S_3$ differ by another NNI move around node 2.

We used BPP to analyze one real and three simulated datasets for Philippine shrews (genus *Crocidura*), published and analyzed previously by Giarla and Esselstyn (2015). Those authors sequenced ultra-conserved elements (UCEs) from 19 individuals representing 7 species of Philippine shrews: *C. palawanensis* (Pl), *C. beatus* (B), *C. mindorus* (M), *C. grayi* (G), *C. panayensis* (Pn), *C. negrina* (Ne), and *C. ninoyi* (Ni), as well as an Indonesian outgroup species, *C. orientalis* (O) (Fig. 8). They generated 1112 UCEs, but 193 of them contained no parsimony-informative sites and were excluded, leaving 919 loci in the dataset. There are up to 19 sequences at each locus, the alignment length ranges from 232 to 1069 sites among loci (with median 706), and the number of parsimony-informative sites ranges from 1 to 17 (with median 2). The authors’ phylogenetic analysis suggested that a specimen from *C. ninoyi* represented a new species and is treated as a distinct species in the analysis, *C. sp* (S). To evaluate the reliability of species tree estimation, Giarla and Esselstyn (2015) simulated 3 datasets by using parameter estimates under the MSC ($\tau$s and $\theta$s) obtained from the UCE data. These are referred to as Sim1-Matching, Sim2-Multi, and Sim3x, each having 500 loci and 700 bp in the alignment per locus. Sim1-Matching matches the characteristics of the UCE dataset, with 19 sequences per locus. Sim2-Multi includes five sequences per species (with 45 sequences per locus) and was used to assess the effect of increased sequence sampling. Sim3x was generated by increasing the $\tau$s by 3-fold while keeping the $\theta$s unchanged, and was used to examine the impact of increased mutation rate and increased phylogenetic information per locus. Giarla and Esselstyn (2015) used 4 methods of species tree estimation to analyze each of the 4 datasets (UCE and 3 simulated datasets): (i) concatenation with MrBayes, two summary coalescent-based methods: (ii) MulRF (Chaudhary et al. 2013) and (iii) ASTRAL (Mirarab and Warnow 2015), and (iv) the Bayesian coalescent method *BEAST (Heled and Drummond 2010). For *BEAST, Giarla and Esselstyn (2015) divided each of the four datasets into subsets of 50 loci, as they observed “little evidence of convergence in analyses of more than 50 loci in *BEAST, even after billions of MCMC generations.”

Here we analyze the 4 datasets using the new version of BPP. Giarla and Esselstyn (2015) used model testing to identify the appropriate substitution model for every locus and encountered numerical problems as the data lack information to estimate the rate parameters in the parameter-rich models such as the GTR (Tavare 1986; Yang 1994). We bypassed this mechanical process of model selection and used JC69 (Jukes and Cantor 1969) throughout. The main role of the mutation/substitution model in such analysis is to correct for multiple changes at the same site to extract information about the gene tree topology and branch lengths at every locus. For such highly similar sequences, JC69 should be adequate (Satta et al. 2004; Burgess and Yang 2008). For datasests Sim1, Sim2, and UCE, we assign the gamma prior $\tau_0 \sim G(2, 1000)$, with mean 0.002 (two mutations per kb) for the age of the root of the species tree (Yang and Rannala 2010, Equation 2), and $\theta \sim G(2, 1000)$ for all $\theta$ parameters. For Sim3x, we used $\tau_0 \sim G(2, 300)$ and $\theta \sim G(2, 1000)$. We used a burn-in of 32,000 iterations, and took $10^5$ samples, sampling every 4 iterations. For each analysis of the simulated datasets, the program was run 3 times using different starting species trees. Each run (on a single core) took about 1 day for Sim1 and Sim3, and 3–4 days for Sim2. Longer chains were run for the UCE dataset, as reported below.

#### Dataset Sim1-Matching (500 loci).

The three BPP runs using different starting species trees produced the same MAP tree (Fig. 8a), with the posterior probability varying from 0.42 to 0.46 among the runs, indicating that the 3 chains have converged to the equilibrium distribution. The 3 MCMC samples were then merged and summarized. The MAP tree from the combined sample, which is also the majority-rule consensus tree, had the posterior 45% (Fig. 8a). This is also the true species tree used to simulate the dataset by Giarla and Esselstyn (2015). The posterior for the nodes are shown in Figure 8a. The top 3 species trees are different resolutions of the 3 clades, S, B, and PNN, where PNN stands for the ((Pn, Ne), Ni) clade. Their posterior probabilities are 45% for (S, (B, PNN)) (i.e., the true species tree $S_1$ of Fig. 8a), 22% for ((B, S), PNN), and 19% for ((S, PNN), B), so that those 3 trees constitute the 85% credible set. In the analysis of the same data by Giarla and Esselstyn (2015, Figs. 3–6), all the 4 methods the authors used, including concatenation/MrBayes, MulRF, ASTRAL, and *BEAST, inferred at least one incorrect node, whereas concatenation produced the posterior 100% for the wrong tree. Thus BPP is the only method that recovered the true species tree for this dataset, although the support for two nodes is low. The posterior means and 95% highest posterior density (HPD) intervals for the node ages ($\tau$s) obtained from the BPP analysis are shown in Figure 8a. The posterior means are close to the true values used in the simulation.

#### Dataset Sim2-Multi (500 loci).

The 3 BPP runs using different starting species trees converged to the same neighborhood of the species tree space, visiting 3 species trees that correspond to different relationships among the 3 clades, S, B, and PNN in species tree $S_1$ (Fig. 8a). The posteriors for the 3 resolutions were about 40% for (S, (B, PNN)), which is the true tree $S_1$, 40% for (B, (S, PNN)), and 20% for ((B, S), PNN). In other words, node 1 in the true tree $S_1$ is not well-resolved, whereas all other nodes are recovered with posterior probability $\sim 100\%$. Compared with Sim1, for which the MAP tree has two uncertain nodes, the increased sequence sampling (to five sequences per species) in Sim2 helped to resolve one of the two uncertain nodes, but the other remains unresolved. We note that all 4 methods used by Giarla and Esselstyn (2015, Figs. 3–6), including concatenation, MulRF, ASTRAL, and *BEAST, inferred at least one incorrect node for this dataset, with concatenation to be the only method that gave a 100% support for the wrong tree. In the BPP analysis, the true species tree is one of the top two nearly equally good trees.

#### Dataset Sim3x (500 loci).

The 3 runs using different starting species trees converged to the same MAP tree, which is the true tree $S_1$ (Fig. 8a). The posterior ranged from 0.996 to 0.999 among the 3 runs, and was 0.997 in the combined sample. In this dataset, all 4 methods used by Giarla and Esselstyn (2015) produced the true tree as the estimate. By increasing the node ages by three folds and keeping the population sizes unchanged, the impact of ancestral polymorphism and incomplete lineage sorting is reduced while the sequences at every locus are more divergent and contain more phylogenetic information. As a result Sim3x is far more informative than Sim1.

#### The real UCE dataset (919 loci).

With 919 loci and with many ambiguity characters in the sequence alignments, the UCE dataset was found to be more challenging to analyze than the 3 simulated datasets. Indeed our analysis of this dataset using BPP was not entirely successful, and the program showed mixing problems. We report our analysis of this dataset nevertheless, partly to illustrate the computational difficulties encountered and the techniques for their diagnosis. We conducted 6 initial runs with different (and poor) starting species trees, with a burn-in of 32,000 iterations, and took $10^5$ samples, sampling every 10 iterations. Those runs suggested that 6 species trees had substantial posterior probabilities. Three of them were different resolutions around node 1 in species tree $S_2$ (Fig. 8b) concerning the relationships among the clades B, S, and PNN, whereas the other three were different resolutions around node 1 in species tree $S_3$ (Fig. 8c) concerning the relationships among the clades S, PNN, and MG, where MG stands for the (M, G) clade. We then conducted 6 further runs, using those 6 (good) species trees as the starting tree, and the same settings otherwise. Each run (on a single core) took 6–10 days.

The 3 runs that started using the different relationships of S, B, and PNN in $S_2$ (Fig. 8b) produced similar results, visiting the 3 species trees that correspond to different resolutions of node 1 in $S_2$. The MAP tree was $S_2$: (B, (S, PNN)), and the posterior ranged from 70–77% among the three runs. The three samples combined gave the relative proportions 73% for $S_2$: (B, (S, PNN)), 24% for $S_1$: (S, (B, PNN)), and 3% for ((S, B), PNN).

The 3 runs that started with the different relationships among S, PNN, and MG in species tree $S_3$ (Fig. 8c) produced similar results among them, visiting the 3 species trees that are different resolutions of node 1 in $S_3$. The MAP tree had the relationship $S_3$ (Fig. 8c): (MG, (S, PNN)), and the posterior ranged from 71–80% among the runs. The 3 samples combined gave the relative posteriors 76% for $S_3$: (MG, (S, PNN)), 21% for $S_{3a}$: (PNN, (S, MG)), and 3% for $S_{3b}$: (S, (MG, PNN)).

However, BPP had difficulty moving between the two sets of species trees. Note that species trees $S_2$ and $S_3$ differ by a single NNI move (around node 2 in either tree, Fig. 8b and c), and should be reachable by both SPR and NodeSlider moves. We leave it to future research to investigate the precise reasons for the failure of the chains to move between the two sets of trees and to design improved algorithms. The mixing problem means that the MCMC runs reported here cannot be used to estimate the posterior probabilities for the 6 species trees, or to decide whether $S_2$ or $S_3$ has the highest posterior and is the MAP tree.

We applied the algorithm of Equation (15) to calculate the log marginal likelihood values for species trees $S_2$ and $S_3$, using $K = 16$ Gauss–Legendre points. For each of the 16 MCMC runs to approximate the power posteriors, we use 8000 iterations for the burnin, after which we take $5 \times 10^5$ samples, sampling every 2 iterations. Each run took 4–6 days on a single core. The average log likelihood over the MCMC sample is plotted in Figure 7. This calculation gave the log marginal likelihood ratio $\log f(X|S_2) - \log f(X|S_3)$ or log Bayes factor to be $-3$, which translates to the posterior probability ratio $\textrm{e}^{-3} = 0.050$. This posterior probability ratio can be used to convert the relative posteriors calculated from the MCMC runs discussed above into (absolute) posterior probabilities for the 2 sets of species trees. The posteriors for the top 4 species trees were then $76\% \times \frac{1}{1+\tt{e}^{-3}} = 72\%$ for $S_3$: (MG, (S, PNN)), 20% for $S_{3a}$: (PNN, (S, MG)), 3% for $S_2$ (Fig. 8b) and 3% for $S_{3b}$: (S, (MG, PNN)).

However, we note that the average log likelihood of Figure 7 was poorly estimated by the MCMC sample for the 2 smallest values of $\beta$ (0.0053 and 0.0271), when the power posterior is close to the prior. Our conclusion that species tree $S_3$ is the MAP tree is thus a tentative one. We note that node 2 in $S_3$ has a longer branch than node 2 in $S_2$ (Fig. 8), which supports the notion that $S_3$ is more probable than $S_2$.

Certain phylogenetic relationships are supported in all BPP runs, with 100% posterior, and they were also found in the analyses of (Giarla and Esselstyn 2015). First, among all the species endemic to Philippine, *C. palawanensis* (Pl) diverged the earliest. Second, *C. mindorus* (M), *C. grayi* (G) form a clade. Third, *C. panayensis* (Pn), *C. negrina* (Ne), and *C. ninoyi* (Ni) form a clade, with the relationship ((Pn, Ne), Ni) inside the clade. It is also certain that the MAP tree found in the BPP analysis is different from the best estimate that Giarla and Esselstyn (2015) obtained using alternative methods. As mentioned earlier, the best species tree found by Giarla and Esselstyn was $S_1$. This had a lower posterior probability than $S_2$ in the BPP analysis, while $S_3$ appeared to be the MAP tree.

We also note that the posterior estimates of parameters under the MSC (the $\tau$s and $\theta$s) suggest short internal branches in the species trees (Fig. 8a–c) and small effective population sizes for the ancestors (Fig. 8c; see also Giarla and Esselstyn 2015, Appendix 3). These indicate several radiative speciation events following the colonization of Philippines, with the new species founded by very small populations.

## Discussion

### Statistical Performance of BPP

Our simulation results suggest that the likelihood-based species tree inference method under the MSC, implemented in BPP, has both high precision (as indicated by the small credible set) and high accuracy (as indicated by the high coverage probability of the credible set). For the parameter combinations examined in our simulations, the correct species tree is recovered with high posterior probabilities when 10 loci are included in the dataset. The high power of the method is in contrast to the heuristic coalescent methods which use reconstructed gene tree topologies to infer the species tree, ignoring both random and systematic errors in tree reconstruction and ignoring information in the gene-tree branch lengths, leading to loss of power. Our results are consistent with several previous studies, which suggest that heuristic methods based on summary statistics such as estimated gene tree topologies can be much less efficient than full likelihood methods (Leaché and Rannala 2011; Liu et al. 2015; Ogilvie et al. 2016).

This conclusion is also apparent in the comparison of our BPP analysis of the three simulated datasets of Giarla and Esselstyn (2015) with those authors’ analysis of the same data using four competing methods. Concatenation produced 100% support for every node in the species tree in each dataset, but one of the inferred nodes was incorrect in Sim1 and Sim2 (Giarla and Esselstyn 2015, Fig. 3), so that the high confidence is spurious. Previous simulation and analysis of Kubatko and Degnan (2007) and Roch and Steel (2015) suggest that in certain areas of the parameter space (certain species trees and values of $\theta$s and $\tau$s), concatenation may be statistically inconsistent, converging to an incorrect species tree when the number of loci approaches infinity. The two heuristic coalescent methods, MulRF (Chaudhary et al. 2013) and ASTRAL (Mirarab and Warnow 2015), inferred more nodes incorrectly or produced lower support values for true nodes on the species tree than BPP (Giarla and Esselstyn 2015, Figs. 4 and 5). The *BEAST analysis of Giarla and Esselstyn (2015) used small data subsets of 50 loci, producing many unresolved or uncertain nodes, whereas our BPP analysis used the full datasets, so that the results cannot be used to make a sensible comparison of the statistical performance of the two programs.

### Computational Challenges and MCMC Diagnostics

The computational requirement of Bayesian MCMC methods tends to increase with the increase in the number of species/populations, the number of loci, the number of sequences per locus, and the number of sites per sequence. The number of species may have the greatest impact, because more species mean many more species trees with a much expanded parameter space, whereas the number of sites is the least important. The increased computational effort may manifest itself in two ways. First, with more data, each iteration of the MCMC algorithm takes more computation, mainly because the phylogenetic likelihood (the probability of observing the sequence alignment at the locus given the gene tree and coalescent times) is more expensive. In typical data analysis, the likelihood calculation accounts for most ($> 80\% $) of the CPU time. The likelihood calculation on a gene tree grows roughly linearly with the number of sequences, and less than linearly with the number of sites in the sequence, although more sequences at each locus also imply more gene trees and branch lengths to average over. Second, with more data, the posterior distribution of the parameters ($\tau$s and $\theta$s) under each species tree becomes more highly concentrated, and as a result it becomes more difficult to move from one species tree to another in the transmodel MCMC algorithm, and many more iterations will be necessary to allow adequate sampling of the posterior. If the proposed parameter values for the new species tree are not good, the proposal will be rejected even if the new species tree has a higher posterior probability than the current species tree. This second problem of poor mixing is a far greater challenge than the first problem of more expensive likelihood calculation per MCMC iteration. This article continues our effort in designing smart transmodel moves to improve the mixing efficiency of the MCMC. The superiority of the SPR and nodeslider moves over NNI is that they allow the transition between species trees that were not direct neighbors by the NNI algorithm, which is important especially during the early stage of the MCMC algorithm or when the starting species trees is poor. The empirical rattlesnake and shrew datasets analyzed in this article were found to be beyond the limit of the NNI algorithm we implemented earlier (Yang and Rannala 2014). Roughly speaking, the improvements made in this study appear to have increased the limit of the program from about 20–100 loci (with $\sim 20$ sequences per locus) to about 200–1000 loci.

One caveat to this discussion is the effect of the data size on the posterior probabilities of species trees. If we simulate datasets with the species tree (and the number of species) fixed, increasing the number of loci or the number of sequences per locus (and, to a lesser extent, the number of sites per sequence) will increase the probability of recovering the true tree, so that a single species tree may dominate the MCMC algorithm, with posterior about $100\%$ in every dataset. In such a scenario, multiple runs that start from different species trees may converge quickly to the same species tree, and the computation may be even less problematic than in smaller datasets in which many species trees have substantial posterior probabilities.

Note that the acceptance proportion of cross-tree moves is not a reliable indicator of the mixing performance of the transmodel MCMC algorithm, and an acceptance proportion of $\sim 0$ may not necessarily imply a mixing problem. If the MAP tree has posterior near 100%, the chain should stay in that species tree nearly 100% of the time and all proposals to change the species tree should be rejected. Although a poorly mixing chain may be stuck in one species tree, leading to an acceptance proportion of $\sim 0\%$ as well, the two scenarios can easily be distinguished by running multiple chains with different starting species trees. Indeed we have found that the most effective way of diagnosing a transmodel MCMC algorithm is to run the same analysis multiple times, starting with different species trees and parameter values.

Similarly we suggest that the consistency among multiple runs starting with different species trees and parameter values be used as the major criterion for determining the length of the MCMC run, including the burn-in, the sampling frequency, and the number of samples. If the starting species trees are poor (e.g., a random species tree), it will take a long time for the MCMC algorithm to converge to the posterior distribution, so that a long burn-in is required. Good starting species trees—for example, those generated by concatenation or heuristic species tree methods such as MP-EST (Liu et al. 2010) or ASTRAL (Mirarab and Warnow 2015)—may be used to shorten the burn-in, but one should be aware of the risk of missing species trees with high posterior probabilities. We suggest that in the initial stage of exploratory analysis, very different starting species trees including poor ones should be used with long burn-ins to explore the posterior space. Later analysis may use good starting species trees with relatively short burn-ins to sample extensively from the posterior. This is the strategy taken in our analysis of the rattlesnake and Philippine shrew datasets in this article.

Note that MCMC iterations in different programs are not comparable. For example, MRBAYES and BEAST sample one parameter to update in each iteration, whereas BPP updates all parameters in the model one by one in each iteration, so that one iteration in BPP may be worth $10^3$ iterations in MRBAYES or BEAST. Note also that the effective sample size (ESS) calculated using the log likelihood value is not useful for diagnosing transmodel MCMC algorithms for species tree inference.

### Limitations and Future Work

Besides the computational challenges in handling large datasets, we note two further limitations of our current implementation in BPP. The first is the use of the Jukes and Cantor (1969) mutation/substitution model. Although this appears to be adequate when closely related species are analyzed so that the sequences are highly similar and multiple hits at the same site are rare, the model may not be suitable for analysis of distant species such as different orders of mammals or land plants. It should be straightforward to implement more sophisticated substitution models. The second is the assumption of the molecular clock, which is expected to be seriously violated in comparisons of distantly related species. It is well-known that molecular clock rooting of phylogenetic trees is unreliable when the clock is seriously violated. We note that the relaxed-clock models developed for dating species divergences are designed for species data and should not be used directly to account for rate variation among branches of the gene tree. However, it appears to be straightforward to modify the model for use under the MSC. Instead of assigning a rate for each branch on the gene tree for the locus, we assign a rate for each branch on the species tree for every locus, so that different gene-tree branches residing in the same species should have the same rate.

## Software Availability

The algorithms described in this article are implemented in the program BPP version 3.3, which may be downloaded from http://abacus.gene.ucl.ac.uk/software/. A small C program called BFdriver is written to generate the control files and job submission scripts for the multiple BPP MCMC runs to sample from the power posteriors for calculation of the marginal likelihood (or the Bayes factor). This is included in the release as well, with a tutorial using the frogs dataset of Yang (2015) as an example.
